# (*Z*)-3-[(*E*)-3-Phenyl­allyl­idene]indolin-2-one

**DOI:** 10.1107/S1600536809002037

**Published:** 2009-01-23

**Authors:** Hongming Zhang, Shashidhar Kumar Akubathini, Haribabu Ankati, Ed Biehl

**Affiliations:** a Department of Chemistry, Southern Methodist University, Dallas, TX 75275, USA

## Abstract

The title compound, C_17_H_13_NO, synthesized to be tested for neuroprotective activities, consists of an indoline and a phenyl­allyl­idene unit with a dihedral angle of 9.0 (1)° between the two ring systems. There are two independent mol­ecules in the asymmetric unit which are connected into a dimer by inter­molecular N—H⋯O hydrogen bonds.

## Related literature

For the pharmacological properties of 3-substituted indoline-2-ones, see: Sun *et al.* (2003 ▶); Andreani *et al.* (2006 ▶); Johnson *et al.* (2005 ▶). For the synthesis and neuroprotective activities of a series of 3-substituted indoline-2-one derivatives, see: Balderamos *et al.* (2008 ▶). For the original synthesis of the title compound, see: Elliott & Rivers (1964 ▶). For modified synthetic methods, see: Tacconi & Marinone (1968 ▶); Villemin & Martin (1998 ▶). For the crystal structures of related compounds, see: Zhang *et al.* (2008 ▶, 2009 ▶).
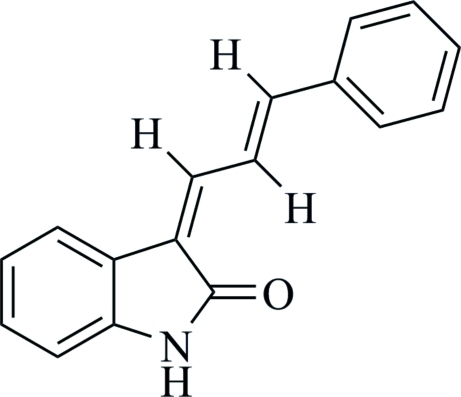


## Experimental

### 

#### Crystal data

C_17_H_13_NO
*M* *_r_* = 247.28
Monoclinic, 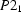

*a* = 5.8373 (4) Å
*b* = 15.3294 (11) Å
*c* = 14.6516 (10) Å
β = 94.312 (1)°
*V* = 1307.35 (16) Å^3^
*Z* = 4
Mo *K*α radiation
μ = 0.08 mm^−1^
*T* = 296 (2) K
0.38 × 0.21 × 0.08 mm

#### Data collection

Bruker SMART APEX diffractometer
Absorption correction: multi-scan (*SADABS*; Sheldrick, 1996 ▶) *T*_min_ = 0.971, *T*_max_ = 0.994
12503 measured reflections
3282 independent reflections
2386 reflections with *I* > 2σ(*I*)
*R*_int_ = 0.032

#### Refinement

*R*[*F* ^2^ > 2σ(*F* ^2^)] = 0.057
*wR*(*F* ^2^) = 0.132
*S* = 1.11
3282 reflections
343 parameters
1 restraint
H-atom parameters constrained
Δρ_max_ = 0.17 e Å^−3^
Δρ_min_ = −0.15 e Å^−3^

### 

Data collection: *SMART* (Bruker 1997 ▶); cell refinement: *SAINT* (Bruker 1997 ▶); data reduction: *SAINT*; program(s) used to solve structure: *SHELXS97* (Sheldrick, 2008 ▶); program(s) used to refine structure: *SHELXL97* (Sheldrick, 2008 ▶); molecular graphics: *SHELXTL* (Sheldrick, 2008 ▶); software used to prepare material for publication: *SHELXTL* and *publCIF* (Westrip, 2009 ▶).

## Supplementary Material

Crystal structure: contains datablocks I, global. DOI: 10.1107/S1600536809002037/bt2843sup1.cif

Structure factors: contains datablocks I. DOI: 10.1107/S1600536809002037/bt2843Isup2.hkl

Additional supplementary materials:  crystallographic information; 3D view; checkCIF report

## Figures and Tables

**Table 1:** Hydrogen-bond geometry (Å, °)

*D*—H⋯*A*	*D*—H	H⋯*A*	*D*⋯*A*	*D*—H⋯*A*
N21—H21⋯O2^i^	0.86	2.03	2.852 (3)	159
N1—H1⋯O22^ii^	0.86	2.07	2.893 (3)	161

Symmetry codes: (i) 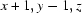; (ii) 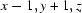.

**Fig. 1.**
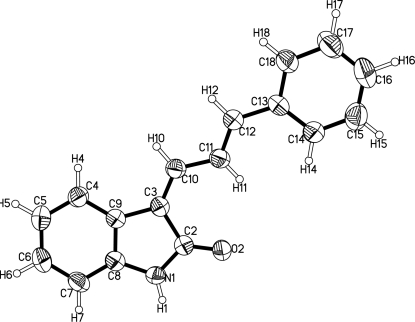
: A view of one of the independent molecules with displacement ellipsoids drawn at the 40% probability level. H atoms are presented as open circles with arbitrary radii. Atoms of another independent molecule were labeled as N21 H21 C22 O22 through C38 H38.

**Fig. 2.**
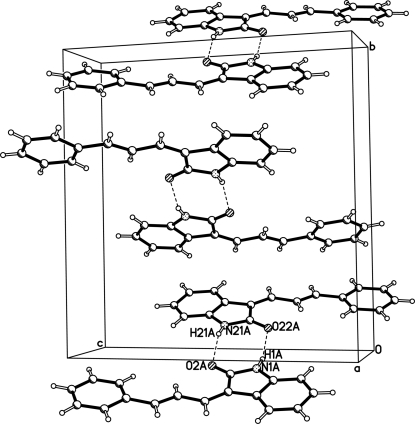
: A unit cell packing view of the title compound. Dash lines indicate hydrogen bonds.

## supplementary crystallographic information

### Comment

It is known that some 3-substituted indoline-2-ones compounds exhibit a variety of pharmacologically important properties such as protein kinase inhibitors (Sun *et al.*, 2003), anti-tumor agents (Andreani *et al.*, 2006) and neuroprotecting agents (Johnson *et al.*, 2005). For studying the biological properties, a series of 3-substituted indoline-2-one derivatives have been synthesized in our lab and their neuroprotective activities have been tested (Balderamos *et al.*, 2008). The results are very promising. To expand our research, a few known compounds were made for test purpose. The title compound was first made by Elliott & Rivers (1964), and modified synthetic methods were reported later (Tacconi & Marinone, 1968; Villemin & Martin, 1998). As a part of our studies on the relationship between the biological activities and solid structures a couple of crystal structures of the derivatives have been carried out (Zhang, *et al.*, 2008, 2009). The title compound consists an indoline and a phenylallylidene unit. The two aromatic rings are slightly twisted with a dihedral angle of 9.0 (1)° (Fig 1). In the crystal the molecules are connected by intermolecular H-bonds between the two independent molecules to form a dimer (Table 1, Fig. 2).

### Experimental

The title compound was synthesized by the condensation of *trans*- cinnamaldehyde (1 mmol) with 2-oxindole (1 mmol) in ethanol (10 ml) in the presence of catalytic amount of piperidine (0.1 mmol). After refluxing for 3 h, the reaction mixture was left to stand for overnight. The resulting crude solid was filtered, washed with cold ethanol (10 ml) and dried. Orange colored single crystals of the compound suitable for *x*-ray structure determination were recrystallized from ethanol.

### Refinement

All H atoms were placed in calculated positions and included in the final cycles of refinement using a riding model, with distances N–H = 0.86 Å and C–H = 0.93 Å, and displacement parameters *U*_iso_(H) = 1.2*U*_eq_(N,*C*). Friedel pairs have been merged prior to refinement.

### Crystal data

**Table:**

C_17_H_13_NO	*F*(000) = 520
*M**_r_* = 247.28	*D*_x_ = 1.256 Mg m^−^^3^
Monoclinic, *P*2_1_	Mo *K*α radiation, λ = 0.71073 Å
*a* = 5.8373 (4) Å	Cell parameters from 3505 reflections
*b* = 15.3294 (11) Å	θ = 2.7–28.1°
*c* = 14.6516 (10) Å	µ = 0.08 mm^−^^1^
β = 94.312 (1)°	*T* = 296 K
*V* = 1307.35 (16) Å^3^	Plates, orange
*Z* = 4	0.38 × 0.21 × 0.08 mm

### Data collection

**Table:**

Bruker SMART APEX diffractometer	3282 independent reflections
Radiation source: fine-focus sealed tube	2386 reflections with *I* > 2σ(*I*)
graphite	*R*_int_ = 0.032
Detector resolution: 83.33 pixels mm^-1^	θ_max_ = 28.3°, θ_min_ = 1.4°
φ and ω scans	*h* = −7→7
Absorption correction: multi-scan (*SADABS*; Sheldrick, 1996)	*k* = −19→20
*T*_min_ = 0.971, *T*_max_ = 0.994	*l* = −19→19
12503 measured reflections	

### Refinement

**Table:**

Refinement on *F*^2^	Primary atom site location: structure-invariant direct methods
Least-squares matrix: full	Secondary atom site location: difference Fourier map
*R*[*F*^2^ > 2σ(*F*^2^)] = 0.057	Hydrogen site location: inferred from neighbouring sites
*wR*(*F*^2^) = 0.132	H-atom parameters constrained
*S* = 1.11	*w* = 1/[σ^2^(*F*_o_^2^) + (0.0629*P*)^2^ + 0.0136*P*] where *P* = (*F*_o_^2^ + 2*F*_c_^2^)/3
3282 reflections	(Δ/σ)_max_ < 0.001
343 parameters	Δρ_max_ = 0.17 e Å^−^^3^
1 restraint	Δρ_min_ = −0.15 e Å^−^^3^

### Special details

**Table:**

Geometry. All e.s.d.'s (except the e.s.d. in the dihedral angle between two l.s. planes) are estimated using the full covariance matrix. The cell e.s.d.'s are taken into account individually in the estimation of e.s.d.'s in distances, angles and torsion angles; correlations between e.s.d.'s in cell parameters are only used when they are defined by crystal symmetry. An approximate (isotropic) treatment of cell e.s.d.'s is used for estimating e.s.d.'s involving l.s. planes.
Refinement. Refinement of *F*^2^ against ALL reflections. The weighted *R*-factor *wR* and goodness of fit *S* are based on *F*^2^, conventional *R*-factors *R* are based on *F*, with *F* set to zero for negative *F*^2^. The threshold expression of *F*^2^ > σ(*F*^2^) is used only for calculating *R*-factors(gt) *etc*. and is not relevant to the choice of reflections for refinement. *R*-factors based on *F*^2^ are statistically about twice as large as those based on *F*, and *R*- factors based on ALL data will be even larger.

### Fractional atomic coordinates and isotropic or equivalent isotropic displacement parameters (Å^2^)

**Table:**

	*x*	*y*	*z*	*U*_iso_*/*U*_eq_	
N1	0.4059 (5)	0.95163 (18)	0.38623 (17)	0.0543 (7)	
H1	0.2755	0.9773	0.3761	0.065*	
C2	0.5085 (5)	0.9366 (2)	0.4708 (2)	0.0489 (8)	
O2	0.4320 (4)	0.95989 (17)	0.54301 (15)	0.0629 (7)	
C3	0.7271 (5)	0.8900 (2)	0.4579 (2)	0.0472 (8)	
C4	0.8935 (6)	0.8445 (2)	0.3042 (2)	0.0570 (9)	
H4	1.0253	0.8175	0.3303	0.068*	
C5	0.8528 (7)	0.8490 (3)	0.2103 (3)	0.0688 (10)	
H5	0.9586	0.8254	0.1729	0.083*	
C6	0.6563 (7)	0.8883 (3)	0.1715 (2)	0.0710 (11)	
H6	0.6319	0.8903	0.1081	0.085*	
C7	0.4945 (6)	0.9250 (2)	0.2242 (2)	0.0616 (9)	
H7	0.3628	0.9516	0.1976	0.074*	
C8	0.5369 (6)	0.9204 (2)	0.3177 (2)	0.0502 (8)	
C9	0.7351 (5)	0.8807 (2)	0.3587 (2)	0.0462 (8)	
C10	0.8870 (6)	0.8656 (2)	0.5242 (2)	0.0516 (8)	
H10	1.0145	0.8360	0.5057	0.062*	
C11	0.8796 (6)	0.8809 (2)	0.6199 (2)	0.0533 (9)	
H11	0.7472	0.9045	0.6414	0.064*	
C12	1.0558 (6)	0.8623 (2)	0.6795 (2)	0.0561 (9)	
H12	1.1814	0.8356	0.6557	0.067*	
C13	1.0757 (6)	0.8790 (2)	0.7785 (2)	0.0537 (8)	
C14	0.9034 (7)	0.9174 (2)	0.8247 (2)	0.0654 (10)	
H14	0.7671	0.9344	0.7927	0.078*	
C15	0.9330 (8)	0.9305 (3)	0.9174 (3)	0.0809 (12)	
H15	0.8159	0.9563	0.9476	0.097*	
C16	1.1324 (10)	0.9062 (3)	0.9662 (3)	0.0879 (14)	
H16	1.1517	0.9164	1.0289	0.105*	
C17	1.3015 (9)	0.8671 (4)	0.9220 (3)	0.0892 (14)	
H17	1.4350	0.8485	0.9549	0.107*	
C18	1.2756 (7)	0.8550 (3)	0.8289 (3)	0.0686 (10)	
H18	1.3949	0.8302	0.7992	0.082*	
N21	1.0612 (5)	0.08155 (18)	0.53233 (18)	0.0536 (7)	
H21	1.1927	0.0565	0.5416	0.064*	
C22	0.9524 (5)	0.0958 (2)	0.4481 (2)	0.0492 (8)	
O22	1.0232 (4)	0.07199 (16)	0.37516 (15)	0.0594 (6)	
C23	0.7335 (5)	0.1433 (2)	0.4633 (2)	0.0456 (8)	
C24	0.5755 (6)	0.1884 (2)	0.6182 (2)	0.0556 (9)	
H24	0.4423	0.2150	0.5930	0.067*	
C25	0.6208 (7)	0.1840 (2)	0.7120 (2)	0.0613 (9)	
H25	0.5180	0.2084	0.7502	0.074*	
C26	0.8194 (7)	0.1435 (3)	0.7497 (2)	0.0622 (10)	
H26	0.8460	0.1403	0.8130	0.075*	
C27	0.9781 (6)	0.1077 (2)	0.6946 (2)	0.0567 (8)	
H27	1.1115	0.0810	0.7197	0.068*	
C28	0.9305 (6)	0.1132 (2)	0.6021 (2)	0.0482 (8)	
C29	0.7319 (5)	0.1524 (2)	0.5624 (2)	0.0456 (8)	
C30	0.5699 (6)	0.1654 (2)	0.3983 (2)	0.0530 (8)	
H30	0.4411	0.1931	0.4183	0.064*	
C31	0.5698 (6)	0.1515 (2)	0.3022 (2)	0.0528 (8)	
H31	0.7001	0.1279	0.2790	0.063*	
C32	0.3890 (6)	0.1712 (2)	0.2440 (2)	0.0559 (9)	
H32	0.2614	0.1940	0.2699	0.067*	
C33	0.3687 (6)	0.1611 (2)	0.1446 (2)	0.0522 (8)	
C34	0.5387 (7)	0.1255 (3)	0.0959 (3)	0.0687 (10)	
H34	0.6731	0.1050	0.1268	0.082*	
C35	0.5111 (8)	0.1201 (3)	0.0017 (3)	0.0817 (12)	
H35	0.6266	0.0957	−0.0305	0.098*	
C36	0.3149 (9)	0.1503 (3)	−0.0446 (3)	0.0852 (13)	
H36	0.2974	0.1468	−0.1081	0.102*	
C37	0.1474 (8)	0.1852 (3)	0.0019 (3)	0.0829 (13)	
H37	0.0140	0.2058	−0.0297	0.099*	
C38	0.1718 (6)	0.1907 (3)	0.0951 (2)	0.0672 (10)	
H38	0.0538	0.2148	0.1262	0.081*	

### Atomic displacement parameters (Å^2^)

**Table:**

	*U*^11^	*U*^22^	*U*^33^	*U*^12^	*U*^13^	*U*^23^
N1	0.0485 (14)	0.0618 (19)	0.0516 (16)	0.0099 (13)	−0.0044 (12)	−0.0011 (13)
C2	0.0476 (17)	0.0503 (19)	0.0489 (19)	−0.0030 (15)	0.0037 (15)	0.0000 (15)
O2	0.0579 (14)	0.0837 (19)	0.0471 (14)	0.0140 (13)	0.0039 (11)	−0.0010 (13)
C3	0.0504 (18)	0.0450 (19)	0.0457 (19)	−0.0058 (14)	0.0016 (15)	−0.0018 (14)
C4	0.0536 (18)	0.062 (2)	0.055 (2)	0.0036 (17)	0.0005 (16)	−0.0079 (17)
C5	0.074 (2)	0.078 (3)	0.056 (2)	0.000 (2)	0.0158 (19)	−0.0088 (19)
C6	0.087 (3)	0.084 (3)	0.042 (2)	−0.002 (2)	0.0042 (19)	−0.0036 (19)
C7	0.072 (2)	0.062 (2)	0.049 (2)	0.0020 (18)	−0.0097 (17)	0.0047 (17)
C8	0.0557 (18)	0.0449 (18)	0.049 (2)	−0.0053 (15)	−0.0015 (15)	−0.0026 (15)
C9	0.0484 (17)	0.0418 (17)	0.0475 (19)	−0.0043 (15)	−0.0019 (14)	0.0002 (15)
C10	0.0512 (18)	0.052 (2)	0.051 (2)	0.0020 (16)	0.0014 (15)	0.0002 (16)
C11	0.0520 (18)	0.055 (2)	0.052 (2)	0.0042 (16)	0.0013 (16)	0.0052 (17)
C12	0.058 (2)	0.061 (2)	0.050 (2)	0.0075 (17)	0.0041 (16)	0.0026 (17)
C13	0.0572 (19)	0.057 (2)	0.0453 (19)	−0.0013 (17)	−0.0037 (15)	0.0055 (16)
C14	0.070 (2)	0.070 (2)	0.056 (2)	0.0124 (19)	0.0078 (18)	0.0073 (19)
C15	0.097 (3)	0.083 (3)	0.066 (3)	0.005 (2)	0.024 (2)	0.000 (2)
C16	0.113 (4)	0.096 (3)	0.053 (2)	−0.009 (3)	−0.002 (3)	−0.011 (2)
C17	0.089 (3)	0.113 (4)	0.062 (3)	−0.005 (3)	−0.017 (2)	−0.002 (3)
C18	0.062 (2)	0.087 (3)	0.055 (2)	0.002 (2)	−0.0039 (17)	−0.006 (2)
N21	0.0479 (15)	0.0640 (19)	0.0480 (16)	0.0086 (13)	−0.0024 (12)	−0.0002 (14)
C22	0.0486 (17)	0.0484 (19)	0.0497 (19)	−0.0011 (14)	−0.0016 (14)	0.0023 (14)
O22	0.0573 (13)	0.0792 (17)	0.0421 (13)	0.0106 (12)	0.0067 (10)	−0.0029 (11)
C23	0.0466 (17)	0.0457 (18)	0.0442 (18)	−0.0005 (14)	0.0008 (14)	0.0038 (14)
C24	0.055 (2)	0.059 (2)	0.054 (2)	0.0009 (16)	0.0071 (16)	−0.0014 (16)
C25	0.065 (2)	0.068 (2)	0.052 (2)	−0.0025 (18)	0.0134 (17)	−0.0099 (18)
C26	0.083 (2)	0.063 (2)	0.0400 (19)	−0.003 (2)	0.0013 (17)	−0.0005 (17)
C27	0.062 (2)	0.060 (2)	0.0467 (19)	0.0022 (17)	−0.0053 (15)	−0.0006 (17)
C28	0.0514 (18)	0.0470 (18)	0.0459 (18)	−0.0026 (16)	0.0016 (15)	−0.0043 (15)
C29	0.0500 (17)	0.0451 (18)	0.0416 (18)	−0.0027 (15)	0.0026 (14)	−0.0012 (15)
C30	0.0478 (17)	0.059 (2)	0.053 (2)	0.0019 (16)	0.0040 (15)	0.0051 (17)
C31	0.0538 (18)	0.058 (2)	0.0463 (19)	0.0003 (17)	−0.0006 (15)	0.0047 (16)
C32	0.0533 (19)	0.063 (2)	0.052 (2)	0.0010 (17)	0.0059 (16)	0.0080 (17)
C33	0.0563 (19)	0.053 (2)	0.0476 (19)	−0.0018 (17)	0.0057 (15)	0.0025 (15)
C34	0.070 (2)	0.076 (3)	0.060 (2)	0.008 (2)	0.0065 (19)	0.008 (2)
C35	0.090 (3)	0.092 (3)	0.066 (2)	0.010 (3)	0.020 (2)	−0.008 (2)
C36	0.105 (3)	0.104 (3)	0.045 (2)	−0.005 (3)	−0.001 (2)	−0.002 (2)
C37	0.085 (3)	0.105 (3)	0.056 (2)	0.010 (3)	−0.013 (2)	−0.001 (2)
C38	0.063 (2)	0.080 (3)	0.057 (2)	0.0100 (19)	−0.0065 (18)	0.000 (2)

### Geometric parameters (Å, °)

**Table:**

N1—C2	1.355 (4)	N21—C22	1.362 (4)
N1—C8	1.392 (4)	N21—C28	1.407 (4)
N1—H1	0.8600	N21—H21	0.8600
C2—O2	1.232 (3)	C22—O22	1.230 (4)
C2—C3	1.487 (4)	C22—C23	1.501 (4)
C3—C10	1.348 (4)	C23—C30	1.341 (4)
C3—C9	1.464 (4)	C23—C29	1.460 (4)
C4—C5	1.380 (5)	C24—C25	1.380 (5)
C4—C9	1.383 (5)	C24—C29	1.386 (4)
C4—H4	0.9300	C24—H24	0.9300
C5—C6	1.380 (6)	C25—C26	1.392 (5)
C5—H5	0.9300	C25—H25	0.9300
C6—C7	1.383 (5)	C26—C27	1.386 (5)
C6—H6	0.9300	C26—H26	0.9300
C7—C8	1.374 (4)	C27—C28	1.366 (4)
C7—H7	0.9300	C27—H27	0.9300
C8—C9	1.402 (4)	C28—C29	1.394 (4)
C10—C11	1.426 (5)	C30—C31	1.423 (4)
C10—H10	0.9300	C30—H30	0.9300
C11—C12	1.328 (4)	C31—C32	1.341 (4)
C11—H11	0.9300	C31—H31	0.9300
C12—C13	1.469 (4)	C32—C33	1.460 (4)
C12—H12	0.9300	C32—H32	0.9300
C13—C18	1.384 (5)	C33—C34	1.377 (5)
C13—C14	1.386 (5)	C33—C38	1.388 (5)
C14—C15	1.370 (5)	C34—C35	1.380 (5)
C14—H14	0.9300	C34—H34	0.9300
C15—C16	1.371 (6)	C35—C36	1.368 (6)
C15—H15	0.9300	C35—H35	0.9300
C16—C17	1.360 (7)	C36—C37	1.344 (6)
C16—H16	0.9300	C36—H36	0.9300
C17—C18	1.374 (5)	C37—C38	1.365 (5)
C17—H17	0.9300	C37—H37	0.9300
C18—H18	0.9300	C38—H38	0.9300
			
C2—N1—C8	111.8 (3)	C22—N21—C28	111.2 (3)
C2—N1—H1	124.1	C22—N21—H21	124.4
C8—N1—H1	124.1	C28—N21—H21	124.4
O2—C2—N1	124.9 (3)	O22—C22—N21	125.1 (3)
O2—C2—C3	128.2 (3)	O22—C22—C23	128.1 (3)
N1—C2—C3	106.8 (3)	N21—C22—C23	106.7 (3)
C10—C3—C9	128.0 (3)	C30—C23—C29	128.7 (3)
C10—C3—C2	126.5 (3)	C30—C23—C22	125.9 (3)
C9—C3—C2	105.4 (3)	C29—C23—C22	105.2 (3)
C5—C4—C9	118.9 (3)	C25—C24—C29	118.8 (3)
C5—C4—H4	120.6	C25—C24—H24	120.6
C9—C4—H4	120.6	C29—C24—H24	120.6
C4—C5—C6	120.5 (4)	C24—C25—C26	120.5 (3)
C4—C5—H5	119.7	C24—C25—H25	119.7
C6—C5—H5	119.7	C26—C25—H25	119.7
C5—C6—C7	121.9 (3)	C27—C26—C25	121.2 (3)
C5—C6—H6	119.0	C27—C26—H26	119.4
C7—C6—H6	119.0	C25—C26—H26	119.4
C8—C7—C6	117.1 (3)	C28—C27—C26	117.3 (3)
C8—C7—H7	121.4	C28—C27—H27	121.4
C6—C7—H7	121.4	C26—C27—H27	121.4
C7—C8—N1	129.3 (3)	C27—C28—C29	122.8 (3)
C7—C8—C9	122.0 (3)	C27—C28—N21	128.3 (3)
N1—C8—C9	108.7 (3)	C29—C28—N21	108.9 (3)
C4—C9—C8	119.5 (3)	C24—C29—C28	119.3 (3)
C4—C9—C3	133.2 (3)	C24—C29—C23	132.8 (3)
C8—C9—C3	107.2 (3)	C28—C29—C23	107.9 (3)
C3—C10—C11	126.4 (3)	C23—C30—C31	127.7 (3)
C3—C10—H10	116.8	C23—C30—H30	116.1
C11—C10—H10	116.8	C31—C30—H30	116.1
C12—C11—C10	122.2 (3)	C32—C31—C30	122.5 (3)
C12—C11—H11	118.9	C32—C31—H31	118.7
C10—C11—H11	118.9	C30—C31—H31	118.7
C11—C12—C13	127.7 (3)	C31—C32—C33	127.7 (3)
C11—C12—H12	116.2	C31—C32—H32	116.1
C13—C12—H12	116.2	C33—C32—H32	116.1
C18—C13—C14	117.8 (3)	C34—C33—C38	117.4 (3)
C18—C13—C12	118.8 (3)	C34—C33—C32	123.4 (3)
C14—C13—C12	123.4 (3)	C38—C33—C32	119.2 (3)
C15—C14—C13	120.3 (4)	C33—C34—C35	120.5 (4)
C15—C14—H14	119.8	C33—C34—H34	119.8
C13—C14—H14	119.8	C35—C34—H34	119.8
C14—C15—C16	121.1 (4)	C36—C35—C34	120.4 (4)
C14—C15—H15	119.5	C36—C35—H35	119.8
C16—C15—H15	119.5	C34—C35—H35	119.8
C17—C16—C15	119.3 (4)	C37—C36—C35	119.8 (4)
C17—C16—H16	120.4	C37—C36—H36	120.1
C15—C16—H16	120.4	C35—C36—H36	120.1
C16—C17—C18	120.2 (4)	C36—C37—C38	120.4 (4)
C16—C17—H17	119.9	C36—C37—H37	119.8
C18—C17—H17	119.9	C38—C37—H37	119.8
C17—C18—C13	121.3 (4)	C37—C38—C33	121.5 (4)
C17—C18—H18	119.3	C37—C38—H38	119.3
C13—C18—H18	119.3	C33—C38—H38	119.3

### Hydrogen-bond geometry (Å, °)

**Table:**

*D*—H···*A*	*D*—H	H···*A*	*D*···*A*	*D*—H···*A*
N21—H21···O2^i^	0.86	2.03	2.852 (3)	159
N1—H1···O22^ii^	0.86	2.07	2.893 (3)	161

Symmetry codes: (i) *x*+1, *y*−1, *z*; (ii) *x*−1, *y*+1, *z*.
